# School allergy training promotes internal policy review and enhances staff's preparedness in managing pupils with food allergy

**DOI:** 10.1002/clt2.12042

**Published:** 2021-08-04

**Authors:** George Raptis, Rebecca Totterdell, Konstantinos Gerasimidis, Louise Jane Michaelis, Mercedes Perez‐Botella

**Affiliations:** ^1^ Department of Paediatric Allergy and Immunology Royal Hospital for Children Queen Elizabeth University Hospital Glasgow UK; ^2^ Department of Human Nutrition School of Medicine, Dentistry and Nursing University of Glasgow Glasgow UK; ^3^ Department of Immunology, Infectious Disease & Allergy Great North Children's Hospital Newcastle upon Tyne UK; ^4^ School of Community Health and Midwifery, Faculty of Health and Care University of Central Lancashire Preston UK

**Keywords:** anaphylaxis, policy, preparedness, schools, training

## Abstract

**Background:**

Recently non‐statutory allergy management guidance for schools has been produced in the United Kingdom; however, there has been limited progress in implementing this. The aim of this study was to evaluate the effect of face‐to‐face training on self‐reported school staff preparedness in managing the severely allergic child and whether it would stimulate schools' allergy policy review.

**Methods:**

A preparedness survey was conducted prior and 2 months post‐intervention to assess the effect of training on self‐reported preparedness and perceived confidence to manage children with food allergies.

**Results:**

A sample of 18 primary schools that consented to participate were selected. Of the trained schools, 89% of the head teachers felt confident in dealing with an allergy emergency compared to 39% prior training (*p* = 0.016). Post‐intervention all but one had arranged/were considering introducing allergy awareness sessions to help pupils manage their allergies (45% pre‐training vs. post‐training 93%, *p* = 0.003). Preventative measures for accidental exposure to food allergens (i.e., no food sharing policy) were adopted by all (pre‐training 61% vs. post‐training 100%, *p* = 0.03).

**Conclusion:**

A face‐to‐face school allergy training programme enhances self‐reported staff preparedness and promotes internal allergy policy review in managing the needs of these children, hence addressing the current gap between recommendations and practice in schools.

## 1 BACKGROUND

The increased prevalence of allergic diseases in children has now reached epidemic levels and is considered a public health problem.^1^ The extent to which allergy debilitates individual patients, families and society as a whole is often overlooked by those unaffected, yet community preparedness is paramount. Hospital admission rates for anaphylaxis increased by 72% in the last 5 years for those ≤18 years.^2^ Further, up to 20% of anaphylaxis cases occur within school grounds and of these, one in four occurs in pupils not previously deemed at risk.^3^, ^4^

Previously, we reported that school preparedness for anaphylaxis was below the safety standards set by the Department for Education at that time^5^ and those recommended by the European Academy of Allergy and Clinical Immunology.^1^, ^6^ These findings coincide with the previous international research.^7^, ^8^, ^9^, ^10^

When surveyed, school staff expressed a desire for training and a preference for a face‐to‐face format.^6^ This type of training has been shown to elicit a more efficient response to an anaphylaxis scenario, compared with online training^11^ and to be effective at improving school staff awareness and knowledge of allergic diseases.^12^, ^13^, ^14^, ^15^ In comparison, the effect of training on whole school preparedness in the overall management of pupils with severe allergies and in triggering policy review has rarely been studied.^16^, ^17^

UK schools have a statutory duty of care for children with medical needs, for which the Department for Education has published robust guidance.^5^ However, the implementation of the recommended safety measures at school is suboptimal. Gaps in the current management of pupils with severe allergies have led to call for action from a legislative perspective.^18^

This pilot study aimed to evaluate the effect of a face‐to‐face training programme for schools in England, on school's self‐reported preparedness in managing the needs of children living with severe allergies. It also investigated whether the delivery of the training programme stimulated an allergy policy review within the school and a change in attitude towards the management of such pupils.

## 2 METHODS

### 2.1 Participants

Primary and secondary schools across Cumbria, North West of England, UK, were invited to take part in a survey of school preparedness for anaphylaxis between 2015 and 2016. The results of this survey have been reported previously.^6^

Upon completion of the survey, schools were invited to participate in a training intervention in allergy management; 183 schools responded to the survey and also to the training invitation (response rate 58%). Only primary schools (*n* = 157, 57%) were selected for training. Data from secondary school respondents were not selected for training due to the small sample size (*n* = 22). Special needs schools, academies, colleges and nurseries were also excluded. The primary schools that responded and consented to receive training were first stratified into six groups according to their catchment area and an ID number was assigned to each of them. No other information such as registered pupils with allergies or not was available during the selection process. Based on the resources available to the research team, three schools were selected from each group (every fourth school on the list) to make up a convenience sample of 18 schools.

### 2.2 Intervention

Training was arranged after school hours and all staff (including teachers, teacher assistants, administrative, catering and cleaning personnel, bus driver, etc.) were invited to attend.

The material was peer reviewed for its appropriateness by the multi‐professional team of the local allergy services, including a child psychologist, a patient support group representative, community nurses responsible for the school training in the area and paediatric allergists from other centres.

An allergy specialist delivered a 90‐min training session which consisted of a theoretical and a practical workshop. The training included an interactive presentation covering the overall management of the child with severe food allergy and drills in the management of severe allergic reactions/anaphylaxis. The main thematic sections of the training session are presented in Table 1.

**TABLE 1:** Allergy management workshop (90 min)

Theoretical session		
Allergy management awareness presentation (45 min)		
Setting up an allergy management healthcare plan Training for school staff, parents and pupils Allergy and anaphylaxis prevention measures		Seamless communication with all involved Crisis management Psychological aspects of food allergy associated anaphylaxis
Round table; resources and demonstration (15 min)		
Handbook for developing school emergency protocol on anaphylaxis management	A guide, based on national guidelines,^1^, ^19^ peer reviewed and tailored to UK statutory guidance^5^ in how to develop an anaphylaxis management protocol, was offered to the head teachers. Schools were advised to cross‐check their existing emergency protocol with the guide provided.	
Allergy action plans (British Society Allergy & Clinical Immunology)	Advice was offered on the schools' existing allergy care plans	
Emergency bag demonstration	Guidance on the storage of emergency kits in schools, including which medications are required and their labelling, as per published guidance.^1^ A practical demonstration with a highly identifiable bag was conducted.	

**Practical skills session**		
Hands on session (30 min)		
Anaphylaxis management drills	Scenarios on the management of a severe allergic reaction presenting with respiratory difficulties and signs of hypotension (reduced consciousness, collapse, etc.) were used. Training drills demonstrated and explained included: (i) the appropriate positioning of the patient; (ii) the administration of the AAI; and (iii) the role play scenarios of the necessary communication between the school staff during the crisis management period to both emergency services and parents.	
Practical administration of AAI	All school staff attending the training day practised the administration of the AAI through role play.	

*Note:* Resources used from the Anaphylaxis Campaign (AllergyWise Online Training for Healthcare Professionals, https://www.anaphylaxis.org.uk/information‐training/allergywise‐training/for‐healthcare‐professionals/) and the Australasian Society of Clinical Immunology and Allergy (anaphylaxis e‐training for schools and childcare https://etraining.allergy.org.au/) after granted permission for their use.

Abbreviation: AAI, adrenaline autoinjector.

In order to tailor the training programme to schools' needs, this was first delivered to a group of primary school teachers outside the surveyed area. Upon receiving feedback, the training programme was revised to expand on the administration of the adrenaline autoinjector (AAI) (pupil positioning, restraining. etc.).

### 2.3 Post‐training session assessment

Eight weeks after the training, head teachers (or those deputized by the school and who attended the training) were asked to complete the follow‐up questionnaire; a 26‐item, structured questionnaire.

The three main aspects that were surveyed prior to the workshop were surveyed again (presented in Table 2). The questions were designed as dichotomous or Likert‐type scales and free text options were also available for some questions.^6^ For the design of the questionnaire, to collect and transfer the data, the Teleform information capture system (OpenText™) was used. Participants were asked to return the questionnaire within 2 weeks. Those who failed to do so were sent two further reminders and were also telephoned to encourage response.

**TABLE 2:** Follow‐up survey

Follow‐up questionnaire
Areas surveyed:School staff confidence and preparedness Survey of staff's confidence in managing pupils with severe allergies and those with no such history. Allergy management training (training arrangements offered to and by schools) To capture data on training arrangements that have been put in place or are considered for staff as well as for pupils and their families on the management of allergies. Preventative measures To capture any changes in school policy with regard to allergy prevention.

### 2.4 Statistical analysis

The pre and post‐survey responses were analysed in conjunction. In order to assess the school preparedness pre and post‐training, missed responses (min = 1, max = 6, and median = 2) and ‘don't know’ responses (min = 1, max = 5, and median = 1) to the baseline survey questions which were answered in the post‐training survey were considered as negative answers. It was felt that lack of awareness of specific preventative measures, for example, from the senior management team, was likely to indicate that those measures were not in place.

The chi‐square test was used to compare schools who received training with those who returned the survey but were not selected for training. The Mann–Whitney test was used to identify differences in the responses from schools with registered pupils with allergies and those without pre and post‐training. The McNemar test was used to examine whether training improved schools' preparedness, and it was reported as binary outcomes. A value of *p* < 0.05 was considered statistically significant. The IBM SPSS Statistics v22 was used for the analysis.

## 3 RESULTS

The training programme was delivered to 18 primary schools; a total of 191 school personnel, that ranged from 3 to 25 attendees per school (median = 9, interquartile range = 6). Participating schools originated from all six districts in the county. About 44% (8/18) of the schools were from the most densely populated district. All schools were state‐funded and there were of small‐to‐medium size ranging from 29 to 428 pupils (median = 128, SD ± 119).

It seems that schools with registered pupils with food allergies and especially those with the previous episodes of anaphylaxis were most likely to accept the training offered (58 (41.7%) vs. 13 (72.2%), *p* = 0.014). The analysis of the rest of the schools' characteristics (demographics and preparedness level) for both of those which received training and those which were not selected did not differ. With regard to preparedness level, no significant statistical differences were found in their confidence level in the management of anaphylaxis (*p* = 0.3), in the existence of a standard management protocol for allergy emergencies (*p* = 0.4) or in the preventative measures (except for food bans which the majority of the selected schools had already in place prior to training [47 (48%) vs. 14 (82.4%), *p* = 0.009]).

About 29% (5/18) of the schools had pupils at risk of anaphylaxis and carried an AAI; two of these schools (12%) reported that a personalised allergy action plan was not available.

The response rate to the follow‐up survey was 78% (14/18). The schools' characteristics (number of pupils registered, locality, socioeconomic status or size) of non‐respondents did not differ compared to the respondents.

Fewer than half of the head teachers (39%, 7/18) reported confidence in dealing with an allergic reaction at baseline survey. Following the intervention, 86% (12/14) of respondents stated they felt confident if faced with such emergency (*p* = 0.016).

The majority of head teachers (94%, 17/18) reported that they had procedures on both the identification of pupils with allergies on enrolment at school and the reduction of risks and management of allergic reactions (Table 3). Following training, all head teachers, 100% (14/14) reviewed their practice regarding the identification of pupils with allergies on admission and setting up a management plan. While only 45% (8/18) of the respondents reported that they helped pupils to manage their allergies (providing teaching material and practical skills) prior to training, all but one (93%, 13/14) had arranged or considered introducing such teaching sessions following the intervention (*p* = 0.03).

**TABLE 3:** Mangement of children with allergies at school

Question: Does your school ensure adequate management of allergies for individual children by:					
		Respondents, % (*n*)			
		Yes	No	*n*	*p*
Developing specific procedures to identify children with allergies on enrolment?	Pre	94 (17)	6 (1)	18	
	Post	100 (14)	0 (0)	14	1.00
Developing a plan for reducing the risk of allergic reactions and managing them when they occur?	Pre	94 (17)	6 (1)	18	
	Post	100 (14)	0 (0)	14	1.00
Helping pupils manage their allergies (e.g., by providing teaching material and practical skills)?	Pre	45 (8)	55 (10)	18	
	Post	93 (13)	7 (1)	14	***0.03***

*Note:* Data are presented as the percentage and *p*‐values, values significant if p < 0.05. Statistically significant differences pre and post training are indicated in bold, analysed using the Mcnemar test.

Compared with 44% (8/18) of the head teachers who reported that they were prepared to manage a severe allergic reaction in a child with no previous history of allergy at baseline, 93% (13/14) reported so following the intervention (*p* = 0.016) (Table 4).

**TABLE 4:** School preparedness on the management of severe allergic reactions

Question: Has your school prepared for allergy emergencies by:					
		Respondents, % (*n*)			
		Yes	No	*n*	*p*
Setting up communication systems within the school that are easy to use in emergencies?	Pre	83 (15)	17 (3)	18	
	Post	100 (14)		14	0.5
Making sure staff can get to the AAI quickly and easily?	Pre	78 (14)	22 (4)	18	
	Post	100 (14)		14	0.25
Making sure that AAI is used when needed and that someone contacts emergency medical services immediately	Pre	78 (14)	22 (4)	18	
	Post	100 (14)		14	0.25
Identify the role of each staff member in an allergy emergency	Pre	61 (11)	39 (7)	18	
	Post	93 (13)	7 (1)	14	0.13
Preparing for allergic reactions in children without a prior history of allergies	Pre	44 (8)	56 (10)	18	
	Post	93 (13)	7 (1)	14	***0.016***
Documenting the role of the staff to an allergy emergency	Pre	72 (13)	28 (5)	18	
	Post	100 (14)		14	0.13

*Note:* Data are presented as the percentage and *p*‐values, values significant if p < 0.05. Statistically significant differences pre and post training are indicated in bold, analysed using the Mcnemar test.

Abbreviation: AAI, adrenaline autoinjector.

It is of note that 35% (5/14) of the respondents stated that they introduced a standard management protocol for the first time following the training and all schools updated or implemented a standard management protocol (pre‐training 78% vs. post‐training 100%, *p* = 0.25).

Arrangements for regular staff training were in place in the majority of schools (78%, 14/18). However, 50% (9/18) of the head teachers reported not offering in‐depth training (theory and practical skills sessions) for those who had frequent contact with children with severe allergies. In 44% (8/18) of the schools, there were no arrangements in place to offer specialist training for those responsible for the health of these children (in‐house and hospital‐based training delivered by allergy specialists). Post‐training, 93% (13/14) of the head teachers reported that arrangements were made for regular training of all staff (pre‐training 78%, vs. post‐training 93%, *p* = 0.63) and 86% (12/14) offered in‐depth training (pre training 50% vs. post‐training 86%, *p* = 0.57). However, only 57% (8/14) offered specialist training at follow‐up (pre‐training 56% vs. post‐training 57%, *p* = 0.69).

More than one third of the head teachers (39%, 7/18) reported that preventative measures for accidental exposure to food allergens such as a no food sharing policy were not in place prior to the training taking place. Post‐training, all the head teachers reported that they had adopted such a policy (pre‐training 61% vs. post‐training 100%, *p* = 0.03. About 71% (10/14) of the head teachers put in place special supervision for high risk pupils during meal times (pre‐training 56% (10/18), *p* = 0.45). While 78% (14/18) reported initially that they followed a nuts‐free policy, post‐intervention, only 57% (8/14) reported so (*p* = 0.25) (Table 5).

**TABLE 5:** Preventative measures

Questions					
		Respondents, % (*n*)			
		Yes	No	*n*	*p*
Is there guidance for staff handling food on the prevention of anaphylaxis?	Pre	72 (13)	28 (5)	18	
	Post	86 (12)	14 (2)	14	0.63
Is there special supervision for high risk children at eating times?	Pre	56 (10)	44 (8)	18	
	Post	71 (10)	29 (4)	14	0.45
Is there a no food‐sharing policy for children at your school?	Pre	61 (11)	39 (7)	18	
	Post	100 (14)	0 (0)	14	***0.03***
Is there a no eating utensil sharing policy for children in place at your school?	Pre	44 (8)	56 (10)	18	
	Post	57 (8)	44 (6)	14	0.69
Is there a no‐nut policy for children at your school?	Pre	78 (14)	22 (4)	18	
	Post	57 (8)	43 (6)	14	0.25
Have relevant teaching session (i.e., cooking classes) been reviewed, to ensure no potential trigger foods for anaphylaxis are used?	Pre	61 (11)	39 (7)	18	
	Post	71 (10)	29 (4)	14	0.69
Is there a no eating policy on transport and from schools?	Pre	33 (6)	67 (12)	18	
	Post	79 (11)	21 (3)	14	0.07
Is there a protocol on food provided for special activities taking place outside the school?	Pre	72 (13)	28 (5)	18	
	Post	71 (10)	29 (4)	14	1.00

*Note:* Data are presented as the percentage and *p*‐values, values significant if p < 0.05. Statistically significant differences pre and post training are indicated in bold, analysed using the Mcnemar test.

Also, only one third of the head teachers (33%, 6/18) reported to have a ‘no eating policy on transport to and from school’. Following the intervention, the majority (79%, 11/14) reviewed this policy (pre‐training 33% vs. post‐training 79%, *p* = 0.07).

The majority of head teachers (83%, 15/18) expressed the need for national guidelines on the management in school of children with severe allergies at the baseline survey and all of them did so post‐training (100%, 14/14, *p* = 0.63).

Similarly, post‐training, 93% (11/18) schools either agreed or strongly agreed with the generic provision of AAI to be kept at school (pre‐training 61%, 11/18, *p* = 0.125).

Between the groups of schools with registered pupils with allergies and those without, apart from the fact that pre‐training the former were most likely to have a management protocol in place than the latter (*p* = 0.003), there were not statistically significant differences in their responses post‐training.

## 4 DISCUSSION

This pilot study explored whether a training programme would improve school staff's overall self‐reported preparedeness in the management of the child with severe allergies. We moved beyond the focus of other studies (impact of training on school staff and confidence^15^, ^16^, ^17^ and assessed the head teacher's response to policy review and implementation of preventative measures.

The fact that a number of trained schools implemented an emergency management protocol for the first time following the training confirms the value of training programmes in supporting schools with and without registered pupils with allergies.^6^

A key element of the emergency management protocol is the storage and accessibility of the emergency medication.^1^ During the training, staff were encouraged to visit the emergency kit location to assess whether this was the most appropriate should an emergency arise. Post‐training, all schools had reviewed the accessibility of the emergency kit by staff.

Special supervision for children at high risk during meals is one of the fundamental recommendations for schools.^1^, ^19^, ^20^ As a minimum, young children with severe food allergies should be supervised by designated staff member(s) during mealtimes and indoor/outdoor activities.^1^, ^19^ This recommendation was adopted by a significant number of trained schools.

An area of practice which the majority of schools needed to review as a matter of urgency was the food consumption during pupils' transfer. A ‘no eating policy on transport to and from school’ (unless medically necessary) was not in place. Schools seemed to respond to this call; however, further reinforcement is required.

Evidence suggests that the ‘no‐nut’ policy does not offer additional protection as it has not been proved to reduce the antigen exposure. In addition, measures such as a general allergen‐ban on their own are inefficient in preventing anaphylaxis as it is not possible to eliminate all allergenic foods from the school environment.^20^ Instead, holistic approaches to the management of allergies should be encouraged.^1^ Our training helped schools improve this holistic approach and they proceeded to review their ‘no‐nut’ policy.

Similarly, following training, head teachers reported that they had started providing pupils with teaching material and practical skills to self‐manage their allergies. By engaging children as active participants in the management of their allergies, it is hoped that this may lead them to develop adaptive behavioural strategies in responsibility taking and self‐management of their condition.^21^

This study was not designed to capture any improvement that training may have on the psychological impact that is commonly experienced by pupils with food allergy attending school and their parents/carers.^1^, ^20^, ^22^ Pupils with food allergies and their parents/carers were not surveyed before and after the training as the study aimed to assess changes in school's preparedness and approach to pupils with allergies needs instead. However, it would be important and beneficial for future research to seek service users' involvement in the design of such studies and to capture the impact that training interventions could have on pupils with allergies and their parents/carers. Parents/carers' views, especially around children's safety while under the supervision of other caregivers, should be an outcome measure following interventions such as the one carried out in our study.

The head teachers of trained schools also seemed to acknowledge the need for regular and specialised staff training in anaphylaxis. This correlated with the increased number of requests received by the local allergy services following training for further support. However, school nurses, who would be the most suitable group of school staff to receive more specialised allergy training in managing the needs of pupils living at risk of anaphylaxis, have been redeployed to other community posts.^23^

Yearly training and practice drills for all school staff are recommended.^1^, ^5^, ^20^ We have previously reported that schools recognise that there is a lack of standardisation in the management of the pupil with severe allergies and believe that a national policy along with support in implementing this are needed to enhance safety at school.^6^

Several of the requirements for a safe school environment for children with allergies have been set out in detail in the recent published guidance from the Department of Education.^5^ However, very little has been done to support schools in implementing these measures.^18^ We showed that schools require support, guidance and regular training in order to feel confident in managing pupils with allergies. Several head teachers here reported willingness to implement additional measures to improve preparedness and agree with the generic provision of AAI.

The majority of head teachers reported increased confidence and preparedness in managing pupils with allergies following training, even in pupils with no previous history of severe allergic reactions. Retention of knowledge and skills over time were not measured here. It has previously been reported that levels of self‐rated confidence and preparedness remain significant after 4–12 weeks of follow‐ups^14^ and decline 6 months after training.^13^, ^24^ A combination of yearly face‐to‐face training with online training after 6 months has been recommended before.^5^ A clear step‐by‐step ‘manual’ that guides school staff and offers troubleshooting if an issue arises along with face‐to‐face training for the implementation of an allergy policy and emergency protocol are required. This should be generated centrally and made available to all schools for implementation as mandatory. Schools should be able to prove their competency towards a safer environment for pupils with allergies; their performance in this area should be measured yearly and they should receive constructive feedback along with recommendations for those areas of practice that require improvement.

We acknowledge a number of limitations in this study. Due to the sample being small, not all of the areas tested post‐training reached statistical significance; however, a general trend towards improving preparedness was observed. The results would have been strengthened by comparing the intervention group to a control group and by recording changes in self‐reported preparedness over time to assess retention of knowledge. The fact that the school participation was on a voluntary basis comprises another limitation of the study as this could have introduced a selection bias in the schools who agreed to participate. Furthermore, schools with registered pupils with allergies and especially those with the previous episodes of anaphylaxis were most likely to accept the training offered. Lastly, it has been suggested that staff's perceived confidence is a good indicator of the school preparedness in managing severe allergic reactions.^15^ However, self‐reported confidence and preparedness may be an ineffective way of measuring actual preparedness on its own.

## 5 CONCLUSIONS

This face‐to‐face training programme is effective in improving schools' self‐reported preparedness in managing children with severe food allergies. It has also stimulated an allergy policy review within schools to address staff training needs and those of children living with allergies. These two factors can contribute to the fundamental need to improve the safety and quality of life of patients through an allergy aware society.

## CONFLICT OF INTERESTS

George Raptis received research grants, lecture and consultancy fees from Nutricia Ltd, Mead Johnson, Abbott, Mylan, ALK‐Abelló and allergy patient organisation. Rebecca Totterdell is undertaking an industrial PhD funded by Mylan. Konstantinos Gerasimidis received research grants and had speakers/consultancy fees from Nestle, Nutricia and Dr. Falk. Louise Jane Michaelis received research grants for NIHR portfolio clinical trials from Nutricia and Sanofi and lectures for allergy patient organisations. Mercedes Perez‐Botella states no conflict of interest.
